# Kinetics of ^210^Po accumulation in moss body profiles

**DOI:** 10.1007/s11356-017-9659-0

**Published:** 2017-07-12

**Authors:** Magdalena Długosz-Lisiecka

**Affiliations:** Technical University of Lodz, Institute of Applied Radiation Chemistry, Wróblewskiego 15, 90-924 Łódź, Poland

**Keywords:** Biomarkers, Radionuclide distribution, First-order kinetic, Biosorption, ^210^Po and ^210^Pb in the air, *Pleurozium schreberi*, Accumulation rate, Low background spectrometry

## Abstract

Radionuclide concentration analysis of total moss bodies often gave relatively different results than a separate analysis of each different morphological part of the same sample. The dynamics of the transfer of metals by dust uplifted from the soil and another approach, based on the diffusion of the two radionuclides to the moss, have been analyzed. In the proposed model, short- and long-term approaches have been applied. Each part of a moss’s profile can show different radionuclides accumulation ability, including both ^210^Pb and ^210^Po isotopes. A first-order kinetic model has been used for ^210^Po and ^210^Pb transport between three body components of mosses. This mathematical approach has been applied for ^210^Po activity concentration in the air estimation. For relatively clean deep forest region, calculated concentrations were from 17.2 to 43.8 μBqm^−3^, while for urban air concentrations were higher from 49.1 to 104.9 μBqm^−3^.

**Electronic supplementary material:**

The online version of this article (doi:10.1007/s11356-017-9659-0) contains supplementary material, which is available to authorized users.

## Introduction

^210^Pb and ^210^Po are natural radionuclides present in the atmosphere in result of ^222^Rn exhalation from the ground. Both are widely used as markers of various atmospheric processes, and because of the disequilibrium between their activity, concentration in fresh aerosols are often use for aerosol residence time calculation method (Persson and Holm 2011; Papastefanou 2006; Długosz-Lisiecka and Bem 2012). Each year up to around 11 × 10^9^ Bq of ^210^Po can be emitted to the urban air from the local coal power plants in Lodz city, Poland (Długosz-Lisiecka 2015b).

The ^210^Po radionuclide content in fresh outdoor living plants is the result of the adsorption process from atmospheric precipitation and ingrowth from ^210^Pb decay (Eq. 1) (Persson 2014).

1 $$ {}^{210} P{b}_{22.3\kern0.5em \mathrm{year}}\to {}^{210} B{i}_{5.03\kern0.5em \mathrm{days}}\to {}^{210} P{o}_{138\kern0.5em \mathrm{days}} $$

Mosses are common biomarkers mainly used for the quantitative determination of concentrations most spread pollutions (heavy metals, radionuclides) of the atmosphere. Because of their good adsorption capacity, the use of mosses as bioindicators of atmospheric metal or radionuclides deposition has been widely accepted (Agnan et al. 2015; Dołęgowska and Migaszewski 2013; Koz and Cevik 2014; Basile et al. 2001; Boquete et al. 2014).

The accumulation of ^210^Pb and ^210^Po in the moss body is generally characterized by two distinct processes: biosorption from dry or wet precipitation, or intake from soil, followed by internal transport. Firstly, a rapid radionuclides biosorption occurs to give a steady state, which is then followed by slower internal transport within the plant’s body (Basile et al. 2001; Boquete et al. 2014). This second, slow step is considered as decisive for the final total radionuclides uptake by moss cells (Długosz-Lisiecka 2016; Uğur et al. 2003, 2004; Brumelis and Brown 1997; Steinnes 1995; Krmar et al. 2009, 2016; Aleksiayenak et al. 2013). Passive deposition on the external parts of the moss body has also been taken into account (Kłos et al. 2012). It is generally assumed that the ectohydric mosses, represented by *Pleurozium schreberi*, take mineral components mainly from wet and dry deposition, so they are not greatly influenced by the soil composition (Fernández et al. 2005; Gjengedal and Steinnes 1990). Full analysis of the biosorption and accumulation of the metals should also take into account the translocation of the elements from the soil and their internal conduction by elongated cells, which promote the transport of water driven by surface tension (Kłos et al. 2012; Dołęgowska and Migaszewski 2013).

Both isotopes present in the air show different behaviors in the environment, including their transport and accumulation in plants, then can be transported between the annual increments of feather in the moss, and they are partially eluted due to leaching, depending on the meteorological conditions and the seasonal growth of the moss feather. Moreover, internal (vertical) transport of minerals within moss bodies, from leaves to rhizoids and vice versa, can also be enhanced by the presence of old dead tissue. Therefore, one should be aware that the rejection of dead fragments of stems or rhizoids can cause significant errors in the evaluation of the degree of air pollution by this method, thus, affecting the usefulness of moss as a biomarker.

The mathematical description of all the processes that occur in the moss body after absorption of the metals is quite complicated and depends on the speed of various processes. Several papers describe biosorption processes using the linear forms of the Langmuir, Freundlich, and Dubinin-Radushkevich models (Olu-Owolabi et al. 2012). The aim of this study is to use this type of kinetic investigation to identity activity of the ^210^Po in the air. For characterization of the dynamics of the metal bioaccumulation in the moss plant, at three compartment model has been applied. For each compartment, a first-order kinetic equation was used for analyzing the ^210^Pb and ^210^Po radionuclides’ transport within the mosses.

## Material and methods

Samples were collected in two different environments (Fernández et al. 2005). Three samples were collected from different city centers and, for comparison, five samples were collected from unpolluted deep forest. The #1, #2, and #3 samples were collected during the summer, while samples #4 and #5 were taken in winter, all from a deep forest area, for identification seasonal fluctuation of radionuclide uptake from the environment by mosses.

Three remaining samples, #6, #7, and #8, were taken from the three city centers. Samples were dried in room temperature by 2 or 3 days, and the cleaned from grass, tree trunk, or other. The samples of mosses were divided into three parts: stems leaves, stems, and rhizoids (Długosz-Lisiecka and Wróbel 2014). The sample weights ranged from 1 up to a maximum of 2 g. Only two subsamples were prepare for each moss body parts for analysis radionuclides. Various species of the moss have different biosorption dynamic and accumulation ability; therefore, only one type of moss samples *P. schreberi* were object for this study. *Pleurozium* species is wide spread in local environment and has satisfying biomonitor features (Długosz-Lisiecka and Wróbel 2014).

The ^210^Pb activities were determined by gamma spectrometry analysis in anticoincidence mode (Długosz-Lisiecka 2016). After instrumental ^210^Pb analysis, a radiochemical ^210^Po separation technique was applied before counting this radionuclide with α-spectrometry. Each sample was placed in a beaker filled with 2 and 5 ml of concentrated HNO_3_ and HCl, respectively. In order to calculate the ^210^Po separation efficiency, a known activity of ^209^Po isotope (NIST 4326a) was added to each sample as a marker. After digestion, the samples were evaporated and the dry residues were dissolved in 70 ml of 1 M HCl. Prior to the spectrometry measurement, the ^210^Po and ^209^Po present in the solution were separated by spontaneous deposition on silver discs (the average efficiency of deposition was equal 95%). The activities of ^210^Po and ^209^Po were determined using an α-spectrometry system with a PIPS (CANBERRA) detector (Długosz et al. 2010).

**Fig. 1:**
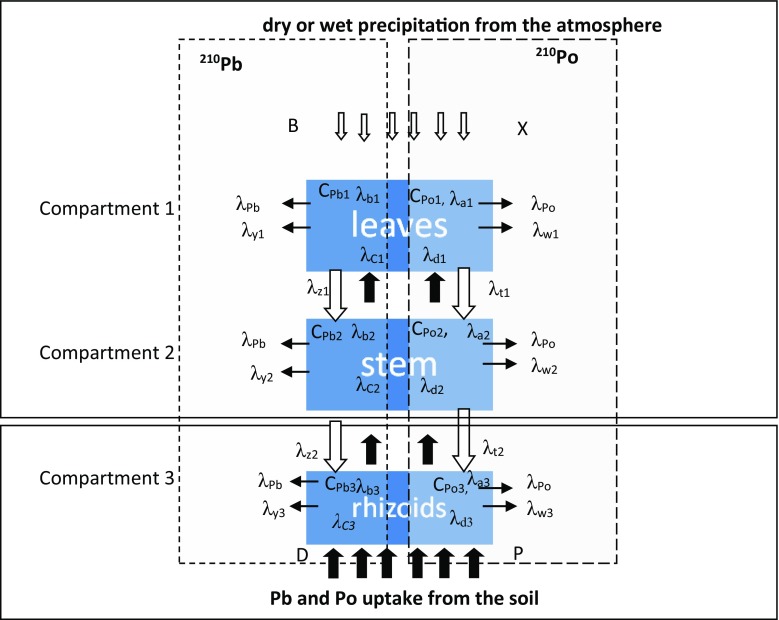
Three compartment models of moss body vertical profile for ^**210**^Pb and ^**210**^Po bioaccumulation

The kinetics of the^210^Po and ^210^Pb radionuclides’ translocation with dust uplifted from the soil was evaluated using the epigeal moss *P. schreberi*. The kinetic parameters for the radionuclide content of the three compartments of the moss are shown in Fig. 1.

Radionuclide uptake kinetics can be described using an adjustment of the three compartment models: Eq. (2, 3, 4) and Eq. (5, 6, 7) for ^210^Pb and ^210^Po, respectively.

2, 3, 4 $$ \left\{\begin{array}{l}\frac{dC_{Pb1}}{dt}={\lambda}_{b1} B+{\lambda}_{c1}{C}_{Pb2}-\left({\lambda}_{y1}+{\lambda}_{Pb}+{\lambda}_{z1}\right){C}_{Pb1}- sB\\ {}\frac{dC_{Pb2}}{dt}={\lambda}_{b2}{C}_{Pb1}+{\lambda}_{c2}{C}_{Pb3}-\left({\lambda}_{y2}+{\lambda}_{Pb}+{\lambda}_{z2}\right){C}_{Pb2}- sB\\ {}\frac{dC_{Pb3}}{dt}={\lambda}_{b3}{C}_{Pb2}+{\lambda}_{c3} D-\left({\lambda}_{y3}+{\lambda}_{Pb}\right){C}_{Pb3}- sB\end{array}\right. $$

5, 6, 7 $$ \left\{\begin{array}{l}\frac{{ d C}_{Po1}}{ d t}={\lambda}_{a1} X+\Delta {C}_{Po1}+{\lambda}_{d1}{C}_{Po2}-\left({\lambda}_{Po}+{\lambda}_{w1}+{\lambda}_{t1}\right){C}_{Po1}- kX\\ {}\frac{{ d C}_{Po2}}{ d t}={\lambda}_{a2}{C}_{Po1}+\Delta {C}_{Po2}+{\lambda}_{d2}{C}_{Po3}-\left({\lambda}_{Po}+{\lambda}_{w2}+{\lambda}_{t1}\right){C}_{Po2}- kX\\ {}\frac{{ d C}_{Po3}}{ d t}={\lambda}_{a3}{C}_{Po2}+\Delta {C}_{Po3}+{\lambda}_{d3} P-\left({\lambda}_{Po}+{\lambda}_{w3}\right)\;{C}_{Po3}- kX\end{array}\right. $$

Where:

C_Po1,2,3;_ C_Pb1,2,3_: activity concentration of ^210^Po and ^210^Pb [Bq kg^−1^] in the moss body, in compartments 1,2, and 3, respectively,
X,B: ^210^Po and ^210^Pb activity concentrations accumulated from the atmosphere [Bq kg^−1^],
λ_a1,2,3,_ λ_b1,2,3,_ ^210^Po and ^210^Pb radionuclides’ accumulation rate (adsorption rate) (day^−1^) for compartments 1,2, and 3, respectively (vertical, down),
λ_t1,2,3;_λ_z1,2,3_: ^210^Po and ^210^Pb elimination rate (desorption) from each compartment of the moss’s body (day^−1^) for compartments 1,2, and 3, respectively,
λ_d1,2,3;_λ_c1,2,3_: ^210^Po and ^210^Pb accumulation rate taking into account transport to the top for each part of the moss’s body (day^−1^) for compartments 1, 2, and 3, respectively (vertical, up),
λ_w1,2,3;_λ_y1,2,3_: ^210^Po and ^210^Pb washout rate from each compartment of the moss’s body (day^−1^) for compartments 1, 2, and 3, respectively, to the outside,
λ_Po,_λ_Bi_: ^210^Po and ^210^Bi radionuclide decay constant [day^−1^],
kX,sB: coefficient resulting from uplifted soil particles deposited on the surfaces of the moss samples for ^210^Po and ^210^Pb, respectively,
ΔC_Po1,2,3_: ^210^Po ingrowth from ^210^Pb decay at time t [days].

As a result, after the integration for a given accumulation time t, a set of equations for each compartment can be obtained. Particularly for leaves (compartment 1), these equations have the following forms (equations for next compartments would have similar form):

8 $$ 1-{ \exp}^{\left(-\left({\lambda}_{y1}+{\lambda}_{z1}+{\lambda}_{Pb}\right) t1\right)}=\frac{\left({\lambda}_{y1}+{\lambda}_{z1}+{\lambda}_{Pb}\right){A}_{Pb1}}{\lambda_{b1} B+{\lambda}_{c1}{A}_{Pb2}- sB}{\mathrm{for}\kern0.5em }^{210}\mathrm{Pb}\ \mathrm{radionuclide} $$

9 $$ 1-{ \exp}^{\left(-\left({\lambda}_{w1}+{\lambda}_{t1}+{\lambda}_{Po}\right) t1\right)}=\frac{\left({\lambda}_{w1}+{\lambda}_{t1}+{\lambda}_{Po}\right){A}_{Po1}}{\lambda_{a1} X+\Delta {C}_{Po1}+{\lambda}_{d1}{A}_{Po2}- kX}{\mathrm{for}\kern0.5em }^{210}\mathrm{Po}\kern0.5em \mathrm{radionuclide} $$

Where:

10 $$ \Delta {C}_{Po1}={A}_{Pb1}\left[\right(1- \exp \left(-{\lambda}_{Bi}{t}_1\right)+\frac{\lambda_{Bi}}{\left({\lambda}_{Bi}+{\lambda}_{Po}\right)}\left( \exp \left(-{\lambda}_{Bi}{t}_1\right)- \exp \left(-{\lambda}_{Po}{t}_1\right)\right)\Big] $$

Both Eqs. (8, 9) show the strong dependence of the A_Pb1_ and A_Po1_ activities on the time of exposure. Both metals are incorporated into the moss’s body (e.g., via adsorption followed by internal transport); therefore, two processes, biosorption and radionuclide transportation, were considered. Firstly, a fast variant of the kinetics, describing the intensive processes of bioaccumulation and washout from compartments, was examined. Secondly, a long-term approach was used, describing the steady state conditions of the dynamics of bioaccumulation, taking into account ^210^Po ingrowth from ^210^Pb decay and its own radioactive decay.

## Results

The levels of ^210^Po and ^210^Pb activity concentration in the various components of the moss’s body depend on several factors, such as the initial content of both radionuclides in the local environment and their activity ratios in the air and soil, along with the total accumulation time, which plays a significant role in the internal transport of metals (Koz and Cevik 2014; Sert et al. 2011; Uğur et al. 2003, 2004). ^210^Pb activity concentration distributions in moss body profiles collected in various environments (forest air, urban air) seemed to be more stable than ^210^Po concentrations. Proposed method focused on the fluctuations of ^210^Po activity concentration distributions in the moss profiles. ^210^Pb distribution analysis at the same profiles is aimed to help in estimation of the real ^210^Po content in the air, only.

### Short-term dynamics of ^210^Po radionuclide bioaccumulation

For a short period of the absorption process (*t* = 0–10 days), ΔC_Po1,2,3_, the ingrowth of ^210^Po values from ^210^Pb decay, has a negligible contribution towards the total estimation of A_Po_ activity and some simplification of the expressions (5–7) can be carried out. In the leaves, all the processes take place at their fastest rate; therefore, the ^210^Po decay constant *λ*_*Po*_ = 0.005 [day^−1^] only gives a small contribution to the exponent value and can be omitted (Ghaemian 1979). It has been assumed, if there is no rain during that time, which mechanically removes the heavy metals from the plant, Eq. 9 that it can be simplified to the form shown below (e.g., for compartment 1). The kX parameter describes the deposition of the uplifted soil particles onto the moss’s surface. Over a short period of time, the kX parameter can be considered to be negligible.

11 $$ 1-{ \exp}^{\left(-\left({\lambda}_{t1}\right) t\right)}=\frac{\lambda_{t1}{A}_{Po1}}{\lambda_{a1} X+{\lambda}_{d1}{A}_{Po2}} $$

Therefore, for a short term (t = <10 days), we can obtain the correlation:$1-{ \exp}^{\left(-\left({\lambda}_{t1}\right) t\right)}->0$

12 $$ {\lambda}_{t1}{A}_{Po1}<<{\lambda}_{a1} X+{\lambda}_{d1}{A}_{Po2} $$

13 $$ {\lambda}_{t1}<{\lambda}_{a1},{\lambda}_{d1} $$

This equation confirms that the uptake of metals from wet or dry deposition seemed to be greater than the amount released from these mosses (Ghaemian 1979). In general, λ_a1_/λ_t1_ exceeds a value of 1.

The ratio of factors, λ_a_/λ_t_, for ^210^Po accumulation in moss describes the speed of two competing processes: accumulation from the atmosphere on the upper layer of the moss’s profile and downwards transport resulting from translocation in different parts of this plant (Olu-Owolabi et al. 2012). The λ_a1_ factor in compartment 1 describes the very effective biosorption of metals by the leaves from the air, whereas -λ_t1_ describes ^210^Po transport down to the stem. Effective ^210^Po sorption at compartment 1 results from well-developed leaf branches and their large surface for the sorption process. The λ_a_/λ_t_ factor ratio is rather typical for each moss species, while also depending on the effective surface of their leaves and local environmental pollution conditions.

### Long-term dynamics of the^210^Po and ^210^Pb radionuclides’ bioaccumulation

Over longer period of time >10 days, the steady-state condition for compartment 1 can be settled and the expression $1-{ \exp}^{\left(-\left({\lambda}_{w1}+{\lambda}_{t1}+{\lambda}_{Po}\right) t1\right)}->1$. In the long term, external and internal transport of ^210^Pb and ^210^Po radionuclides in the moss profile should be taken into account (Eqs. 14, 15).

14 $$ {\lambda}_{w1}+{\lambda}_{t1}+{\lambda}_{Po}={\lambda}_{a1}+{\lambda}_{d1}- k $$

As a result, Eq. 9 has the form:

15 $$ {\lambda}_{a1} X+\Delta {C}_{Po1}+{\lambda}_{d1}{A}_{Po2}- kX=\left({\lambda}_{w1}+{\lambda}_{t1}+{\lambda}_{Po}\right){A}_{Po1} $$

The ΔC_Po1_ parameter, a linear function of ^210^Pb, describes the ingrowth of ^210^Po from ^210^Pb decay. Over a long period (*t* > 1 year) of time, ΔC_Po1_ = A_Pb1_. For the sake of simplicity of calculation, a state of equilibrium for the activity of this parameter has been taken to apply. As a result, Eq. 15 has the simple, linear form *y = ax-b* (Eq. 16).

16 $$ {\mathrm{A}}_{\mathrm{Pb}1}=\left({\lambda}_{w1}+{\lambda}_{t1}+{\lambda}_{Po}\right){A}_{Po1}-{\lambda}_{a1} X-{\lambda}_{d1}{A}_{Po2}+ kX $$

where:

17 $$ a={\lambda}_{w1}+{\lambda}_{t1}+{\lambda}_{Po};\kern0.5em \mathrm{and}\kern0.5em  b={\lambda}_{a1} X+{\lambda}_{d1}{A}_{Po2}- kX $$

where the *x* and *y* coefficients are the ^210^Po and ^210^Pb activity concentrations in the compartments of the vertical moss profile (Eq. 17).

The settling of the steady-state conditions in the moss body takes a longer time in the case of rapid, mechanical, washout processes (Čučulović and Veselinović 2015; Čučulović et al. 2014) and seems to be different for each compartment. In general, saturation conditions cause a slowdown of the accumulation λ_a,_ λ_b_ and λ_t,_ λ_z_ internal transport parameters and change between each of the separate morphological moss parts, for the ^210^Po and ^210^Pb radionuclides. Other processes, such as ^210^Pb and ^210^Po radioactive decay and ^210^Po ingrowth from ^210^Pb, change both the rate and the accumulation process dynamics significantly. These low decay factors λ_Po_ = 0.005 [day^−1^] and λ_Pb_ = 8.5 × 10^−5^ [day^−1^] became essential and should be considered in the long-term process (*t* > 10 days).

On the base of this simple, linear Eq. (15), estimations of the X parameter, which describes the ^210^Po activity concentration in the air, can be applied. Two sets of moss samples, representing two different environments, clean deep forest and city center, were collected. Let us assume the difference in the rate of deposition on the leaves surface and the accumulation in to the moss interior is linearly correlated, and their difference is constant. For simplicity of calculation, the k-*λ*_*a*1_ parameter was set at 0.3 [day^−1^]. The results obtained in taking into account this assumption are show in Table 1.

**Table 1:** Results of estimation of ^210^Po kinetic parameters *X* in [Bq kg^−1^] and [μBq m^−3^], in eight moss profiles (with assumption dust concentration equal 40 μgm^−3^)

Sample no.	Linear equation	*R* ^2^	λ_w1_ + λ_t1_ [day^−1^]	λ_d_ [day^−1^]	*X* [Bq kg^−1^]	*X* [μBq m^−3^]
#1	y = 0.781× + 67.48	0.776	0.77	1.08	813	32.5
#2	y = 0.593× + 101.9	0.999	0.59	0.89	1095	43.8
#3	y = 0.460× + 123.2	0.995	0.46	0.76	778	31.1
#4	y = 0.917× + 110.2	0.994	0.91	1.22	431	17.2
#5	y = 0.856× + 58.61	0.999	0.85	1.16	530	21.2
#6	y = 2.091× − 178.1	0.780	2.08	2.39	2621	105
#7	y = 1.457× − 146.7	0.983	1.45	1.76	2025	81.0
#8	y = 0.958× − 41.05	0.976	0.95	1.26	1226	49.1

There is a linear correlation between ^210^Pb and ^210^Po radionuclides in moss profiles. The ^210^Po and ^210^Pb activity concentrations in the moss profile can be applied for ^210^Po activity concentration estimation in the air. The results presented in Table 1 confirm that there is a significantly higher ^210^Po content in the urban air (samples 6, 7, and 8) than in the clean forest air (1, 2, and 3 for summer, and 4 and 5 for winter).

In relatively non-polluted air, the number of ^210^Po ions attached to particles is low and ranges from dozens to hundreds [Bq kg^−1^], while for polluted regions, the ^210^Po activity concentration attached to the aerosols can reach up to several thousands [Bq kg^−1^], depending on its origin and the particles’ size (Długosz et al. 2010). ^210^Po activity concentration analysis in Bq kg^−1^ units is more profitable for source pollution identification.

In this study, values the ^210^Po activity concentration has been re calculated for the concentration in the air, assuming dust concentration levels equal to 40 μg m^−3^_._ Therefore, in Table 1, X parameter values in μBq m^−3^ units have been also present.

For relatively clean areas, the ^210^Po activity concentration ranges from 23 to 38 μBq m^−3^ for the Arctic (Persson 2014). For comparison, in urban air, its activity concentration varied between 9.44 and 136.9 μBq m^−3^(Długosz-Lisiecka 2015a). X parameter values obtained on the basis of the proposed method are within the range of values provided by other investigators for France, Italy, Germany, and other European countries (Nho et al. 1996; Jia and Jia 2014; UNSCEAR 2000).

**Fig. 2:**
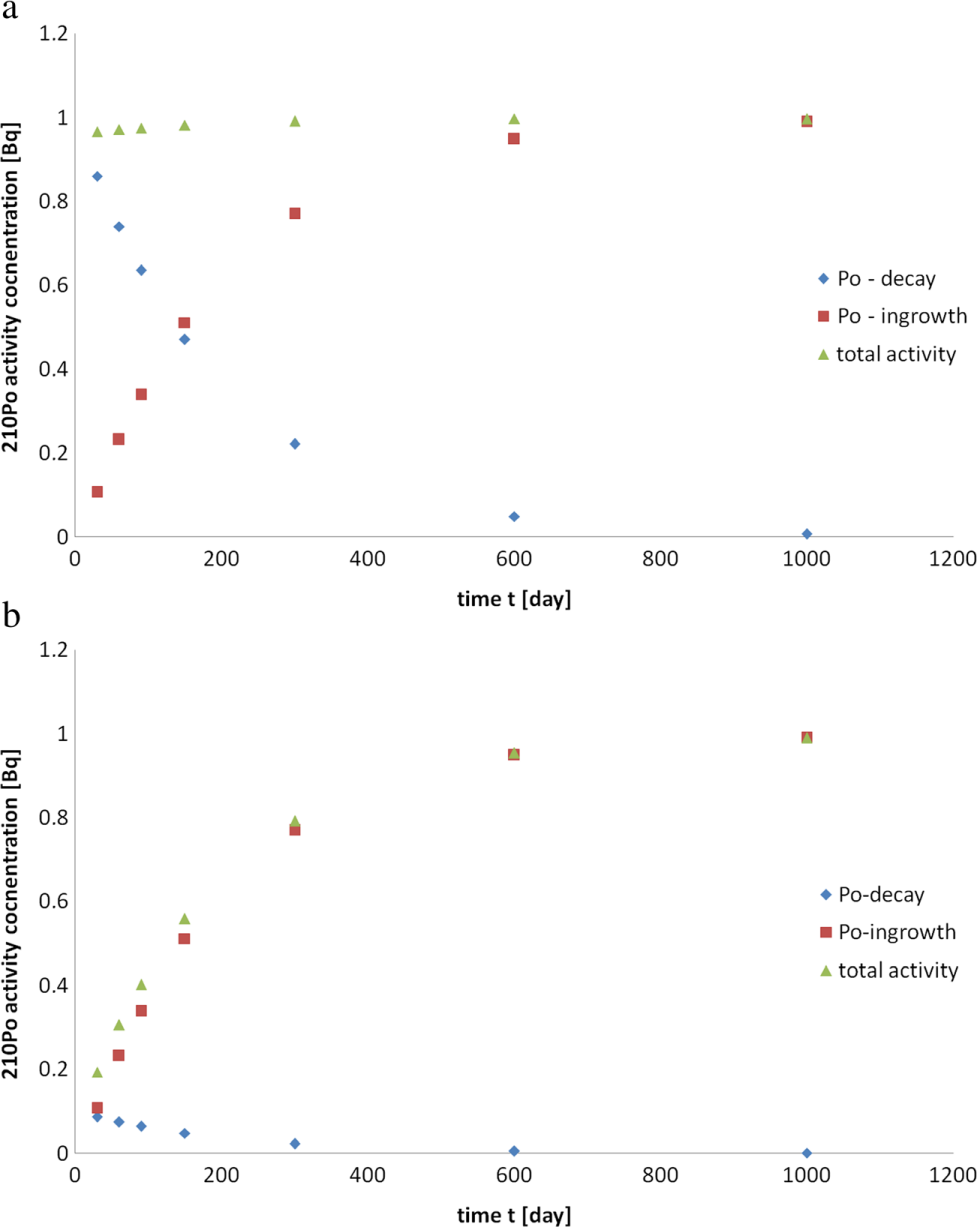
^210^Po activity changes in time **a** ^210^Po/^210^Pb = 1 **b** ^210^Po/^210^Pb = 0.1

In the case of polluted urban environments (air or soil), the activity ratios ^210^Po/^210^Pb ≥ 1 can even exceed unity. As a result, the ^210^Po total activity concentration depends on two processes: ^210^Po decay with a half-life T_1/2_ = 138 days and ingrowth from ^210^Pb (Fig. 2a). If the ^210^Po/^210^Pb initial activity ratio is equal to 0.1 in the different parts of the moss (as is the case for a relatively clean atmosphere), than the ^210^Po content results mostly from ingrowth from ^210^Pb decay (Fig. 2b). The same kinetics between ^210^Po and ^210^Pb radionuclide activities can be obtained for morphological moss parts. However, washout, downward internal transport processes, biosorption, and upward transport processes will all significantly change the dynamic of the steady-state condition.

The results confirmed observations that different fragments of mosses have different contents of ^210^Po and ^210^Pb radionuclides deposited from the local environment. Based on the analysis, it can be concluded that the concentration distributions undergo significant changes with the seasonal variation in their shares of the radionuclides from different emission sources, and the varying transport of minerals within the plant. Solving the first-order kinetic equation for compartment no. 1 (leaves) can give valuable information about the input of fresh atmospheric ^210^Po. Interesting differences have been noticed between samples collected in various locations with different contributions of atmospheric pollution sources. Because of the low number of the sample collected from high polluted regions this study has a preliminary character and will be continue.

## Conclusions

The pollutants accumulated in the leaves of the moss tissues mostly come from atmospheric deposition, rather than from soil contamination. The increased activities of ^210^Po and ^210^Pb in moss body profiles confirm the significant contribution of ^210^Po activity in growth from ^210^Pb decay and aging of the moss tissue. The first-order kinetics of ^210^Po bioaccumulation in each of the morphological moss parts have been used as a method of estimating ^210^Po radionuclide activity concentration in the air.

Based on the radiometric analysis results, one can conclude that the ^210^Po and ^210^Pb concentration distributions depend on seasonal changes in the contributions of different emission sources, as well as the rate of the internal transport of minerals within the plants. The pollutants accumulated in the moss tissues come from sources of atmospheric deposition, rather than from contaminated soil. The disproportion in ^210^Po and ^210^Pb accumulation in different parts of the moss has been measured. Stems and rhizoids can be used for estimation of long-term pollution, while leaves be used for estimation short-term pollution in the air.

## Electronic supplementary material

ESM 1 (DOCX 12 kb)
