# Supplementary material for: Kinetics of 210Po accumulation in moss body profiles
Source: Environ Sci Pollut Res Int. 2017 Jul 12;24(25):20254–60. doi: 10.1007/s11356-017-9659-0 (PMC5574946; doi:10.1007/s11356-017-9659-0)
Supplement: Supplementary file 1 — (DOCX 12 kb) [file 11356_2017_9659_MOESM1_ESM.docx]

**Supplementary material:**

Description of the all procedures and calculations providing information’s about kinetics of ^210^Po and ^210^Pb accumulation in moss profile :

1. Alpha and gamma spectrometry analysis of each moss body parts: leaves, stem and rhizoids.
2. Preparation of the correlation between ^210^Pb and ^210^Po activity concentrations on the base of the three experimental points. In this study for all tested moss samples correlation was linear.
3. On the base of the a and b coefficients ( form of equation y=ax+b) calculate the set of factors: accumulation rate, desorption rate, and other, on the base of equations 14 and 18.
4. Use the results or make some assumptions for estimation of the X factor, which describe ^210^Po activity concentration [Bqkg^-1^] in the dust (equation 17), or [µBqm^-3^] in the air.
